# Physiological Roles and Therapeutic Potential of Ca^2+^ Activated Potassium Channels in the Nervous System

**DOI:** 10.3389/fnmol.2018.00258

**Published:** 2018-07-30

**Authors:** Aravind S. Kshatri, Alberto Gonzalez-Hernandez, Teresa Giraldez

**Affiliations:** ^1^Department of Basic Medical Sciences, Medical School, Universidad de La Laguna, Tenerife, Spain; ^2^Instituto de Tecnologias Biomedicas, Universidad de La Laguna, Tenerife, Spain

**Keywords:** SK channels, IK channels, BK channels, modulators, drug discovery, nervous system, neurological disease

## Abstract

Within the potassium ion channel family, calcium activated potassium (K_Ca_) channels are unique in their ability to couple intracellular Ca^2+^ signals to membrane potential variations. K_Ca_ channels are diversely distributed throughout the central nervous system and play fundamental roles ranging from regulating neuronal excitability to controlling neurotransmitter release. The physiological versatility of K_Ca_ channels is enhanced by alternative splicing and co-assembly with auxiliary subunits, leading to fundamental differences in distribution, subunit composition and pharmacological profiles. Thus, understanding specific K_Ca_ channels’ mechanisms in neuronal function is challenging. Based on their single channel conductance, K_Ca_ channels are divided into three subtypes: small (SK, 4–14 pS), intermediate (IK, 32–39 pS) and big potassium (BK, 200–300 pS) channels. This review describes the biophysical characteristics of these K_Ca_ channels, as well as their physiological roles and pathological implications. In addition, we also discuss the current pharmacological strategies and challenges to target K_Ca_ channels for the treatment of various neurological and psychiatric disorders.

## Introduction

Ca^2+^-activated potassium channels (K_Ca_ channels) constitute a heterogeneous family of ion channels with variable biophysical and pharmacological properties. These channels share a common functional role by coupling the increase in intracellular Ca^2+^ concentration to hyperpolarization of the membrane potential. This intrinsic feature allows K_Ca_ channels to play key roles in controlling cellular excitability and maintaining K^+^ homeostasis and cell volume in non-excitable cells. According to their single-channel conductances, K_Ca_ channels are divided into three main subfamilies: SK (small conductance; ∼4–14 pS), IK (intermediate conductance; ∼32–39 pS) and BK (big conductance; ∼200–300 pS) channels. K_Ca_ channels are expressed in the plasma membrane as tetramers of α subunits. The gene encoding the α subunit (*slo*; *KCNMA1*) of BK channels (KCa1.1; slo1) was cloned in the early 1990s from *Drosophila* (Atkinson et al., 1991; Adelman et al., 1992) and mice (Butler et al., 1993). SKα subunits are products of the *KCNN1* (K_Ca_2.1; SK1), *KCNN2* (K_Ca_2.2; SK2) and *KCNN3* (K_Ca_2.3; SK3) genes (Köhler et al., 1996), whereas IKα (K_Ca_3.1; IK1; SK4) are encoded by the *KCNN4* gene (Ishii et al., 1997b; Joiner et al., 1997). Phylogenetic analysis reveals that K_Ca_ channels belong to two well defined groups (Wei et al., 2005), a division that is paralleled by biophysical properties regarding voltage dependence or the Ca^2+^ sensitivity and regulatory mechanisms.

**FIGURE 1:**
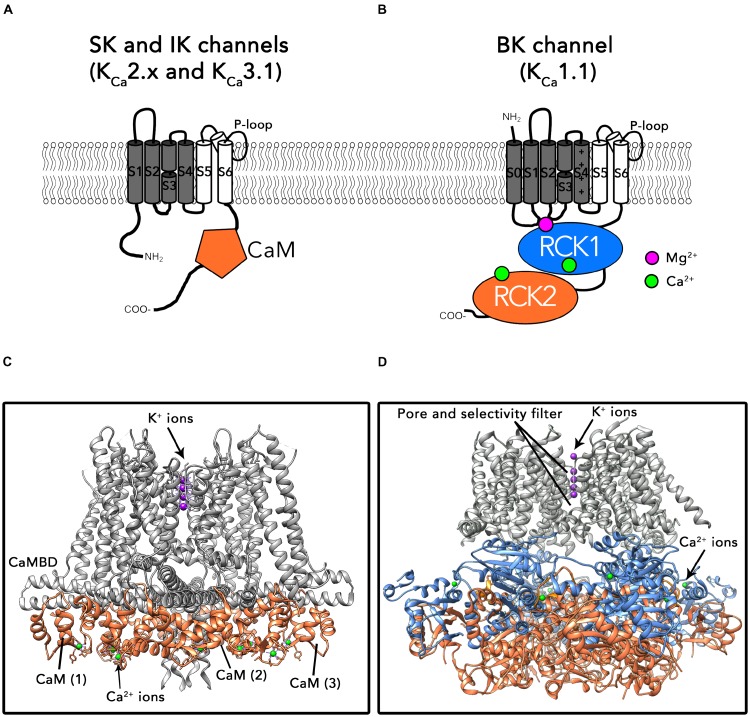
Topology and structures of SK/IK and BK channels. **(A)** Schematic protein topology of one SKα-subunit including the CaM bound to the CaMBD (represented as an orange hexagon). **(B)** Full-length structure of the Ca^2+^-CaM bound IK channel (PDB: 6CNN). The CaM N-lobe binds Ca^2+^ ions after association of CaM to the SK channel C-lobe. **(C)** Schematic topology of one BKα-subunit. Binding sites for divalent cations are located in the cytosolic C-terminal region of the channel. Each α subunit contains two high affinity Ca^2+^ binding sites (represented as green circles) and one low affinity Ca^2+^ and Mg^2+^ binding site, formed by residues from RCK1, S0–S1 and S2–S3 intracellular loops (purple circle). **(D)** Ca^2+^-bound BKα homotetramer full-length structure from *Aplysia californica* (PDB: 5TJ6).

The first group comprises SK and IK channels. As shown in **Figure 1A**, α subunits of these channels consist on six transmembrane helices (S1–S6; the pore region is formed by helices S5 and S6) and cytosolic N- and C-terminal domains. One of their biophysical characteristics is that their function is independent of membrane voltage (Hirschberg et al., 1999). These channels are activated by low intracellular Ca^2+^ concentrations (≈ 0.5 μM) through a mechanism consisting on the existence of a calmodulin (CaM) binding-domain (CaMBD) within the channel’s protein (Fanger et al., 1999; Adelman, 2016). The C-lobe region of CaM is associated to the CaMBD domains following a 1:1 stoichiometry, in a Ca^2+^-independent manner (Lee and MacKinnon, 2018) (**Figure 1B**). Binding of Ca^2+^ to the N-lobes of CaM forces the CaM-CaMBD monomers to arrange structurally into a “dimer of dimers” conformation, pulling the bundle-crossed helices of the pore to open the channel (Schumacher et al., 2001; Lee and MacKinnon, 2018).

BK channels belong to the second group in the K_Ca_ family (for a recent review, see Latorre et al., 2017). The topology of BK α subunits differs from the SK/IK group in the existence of an additional transmembrane helix S0 which drives the N-terminus to the extracellular side of the plasma membrane (**Figure 1C**). The large cytosolic C-terminal region of these channels is composed of two non-identical “regulator for conductance of potassium” domains (RCK1 and RCK2), each one containing a high affinity Ca^2+^ binding site (K_d_ = 0.8–11 μM; (Schreiber et al., 1999; Xia et al., 2002; Sweet and Cox, 2009). In the tetramer, four RCK1-RCK2 tandems form a structure known as the “gating ring” (Wu et al., 2010; Yuan et al., 2011; Hite et al., 2017; Tao et al., 2017) (**Figure 1D**). An additional regulatory site located at the interface between the gating ring and transmembrane region exhibits millimolar affinity for divalent cations such as Mg^2+^ (**Figure 1D**) (Shi et al., 2002; Xia et al., 2002; Yang et al., 2008). In contrast to SK and IK channels, BK are allosterically activated by both changes in the membrane voltage and intracellular Ca^2+^ (Horrigan and Aldrich, 2002). Similarly to other voltage-gated ion channels, the membrane voltage is sensed by charged amino acids within segments S2, S3, and S4 in the transmembrane region (Diaz et al., 1998; Ma et al., 2006; Lee and Cui, 2010; Tao et al., 2017). In contrast to SK and IK channels, Ca^2+^ directly activates BK channels by binding to the specific Ca^2+^ binding sites in the gating ring region, without involving CaM. Binding of Ca^2+^ results on structural rearrangements of the gating ring region, ultimately leading to pore opening (Yusifov et al., 2008, 2010; Lee and Cui, 2010; Javaherian et al., 2011; Yuan et al., 2011; Savalli et al., 2012; Hoshi et al., 2013). It has been proposed that the interaction of the gating ring with the voltage-sensing region of the channel plays an important role in this process (Hite et al., 2017; but see also Zhou et al., 2017) Importantly, the biophysical properties of K_Ca_ channels such as the voltage and Ca^2+^ sensitivities as well as their pharmacological characteristics are greatly influenced by the co-assembly with auxiliary subunits (e.g., β_1-4_, γ_1-4_; see below).

Members of the two groups comprising the K_Ca_ channels family also differ in their structural features. Available crystal structures of the SK CaMBD region bound to CaM have contributed to advance our knowledge about the regulation of these channels by various effectors (Schumacher et al., 2001, 2004; Zhang et al., 2012a,b, 2013; Zhang M. et al., 2014; Nam et al., 2017). Until very recently, structural information about BK channels was the most extensive, including X-ray crystal structures of the isolated C-terminal region known as the “gating ring” (Wu et al., 2010; Yuan et al., 2011) as well as cryo-EM structures of the full-length channel obtained in the presence and absence of Ca^2+^ (Hite et al., 2017; Tao et al., 2017; see also Giraldez and Rothberg, 2017; Zhou et al., 2017) (**Figure 1D**). Exciting new results from the MacKinnon laboratory bring now to light the cryo-electron microscopy (cryo-EM) structures of a human IK-CaM complex in closed and activated states, unveiling unprecedented insights about their gating mechanism (Lee and MacKinnon, 2018) (**Figure 1B**).

The SK, IK, and BK subfamilies show distinct tissue distribution and pharmacology. SK channels are predominantly expressed in the nervous system and are typically inhibited by the bee venom apamin (Park, 1994; Stocker and Pedarzani, 2000). IK channels are mainly distributed in blood, epithelial cells and in some peripheral neurons and are sensitive to the antifungal drug clotrimazole (Gardos, 1958; Jensen et al., 1998). BK channels are the most diverse group of K_Ca_ channels. They are ubiquitously expressed and sensitive to iberiotoxin, charybdotoxin, and paxilline (Latorre et al., 2017). The diversity of K_Ca_ physiological roles is widened by the existence of numerous splicing variants (specially for BK and SK channels) in different tissues (Navaratnam et al., 1997; Shipston, 2001; Chen et al., 2005; Scholl et al., 2014), as well as their association with auxiliary subunits. In vertebrates, BK channels co-assemble with modulatory β (β_1-4_) and γ (γ_1-4_) subunits to modify the channel functional properties and pharmacology (Latorre et al., 2017). In addition, K_Ca_ channels in neurons are closely associated to Ca^2+^ sources such as voltage-gated Ca^2+^ channels (VGCC) (Marrion and Tavalin, 1998; Tonini et al., 2013; Rudolph and Thanawala, 2015; Vivas et al., 2017), transient receptor potential vanilloid (TRPV) channels (Wu et al., 2013; Feetham et al., 2015) and *N*-methyl-D-aspartate receptors (NMDAR) (Isaacson and Murphy, 2001; Ngo-Anh et al., 2005) allowing the precise regulation of membrane excitability and spike firing patterns.

Mutation of genes encoding K_Ca_ channels and subsequent loss- or gain-of-function of these channels has been directly linked to a wide range of human diseases (**Table 1**). Over the last two decades, numerous studies have contributed to decipher the potential role of K_Ca_ channels in these diseases at the molecular level, paving the way for novel therapies. However, despite our current understanding about tissue specific distribution, functional diversity and clinical impact of K_Ca_ channels, they still remain underexploited as targets for drug discovery. In this review, we summarize the most recent knowledge about K_Ca_ channels, focusing on their potential as therapeutic targets in the nervous system and analyzing the factors that could contribute to the difficulties to develop novel K_Ca_ channel modulators. Some excellent reviews in the last few years relate to individual members of the K_Ca_ family, or are centered on particular aspects of K_Ca_ physiopathology (Faber, 2009; Lam et al., 2013; Gueguinou et al., 2014; Contet et al., 2016; Sugunan et al., 2016). Here we initially describe the functional roles of K_Ca_ channels in the nervous system, including their protective roles. Subsequently, the pathologies associated to malfunction of these channels are detailed. Known activators and inhibitors of K_Ca_ channels are presented in the third section of the review, as they may constitute the molecular basis of potential therapeutic strategies to treat a wide range of neurological disorders. Finally, we discuss the possible reasons why little progress has been made toward development of such strategies despite intense research efforts in academia and industry.

**Table 1:** Mutations in K_Ca_ channels that are associated in human diseases, their location in the channel protein, functional implications and related references.

Gene/protein	Disease phenotype	Mutation/location in the protein	Functional effects	Reference
*^∗^KCNN3*/SK3	Idiopathic non-cirrhotic portal hypertension	V450L/intracellular loop between the S4 and S5 transmembrane segments	*De novo* mutation	Koot et al., 2016
			Functional effects currently unknown.	
*KCNN3*/SK3	Schizophrenia	hSK3Δ (*L283fs287X* mutation)/N-terminal cytoplasmic region (deletion)	Suppresses SK currents Disrupts SK-NMDAR coupling	Miller et al., 2001; Soden et al., 2013
*^∗^KCNMB1*/BKβ1	Diastolic hypertension	E65K/Extracellular loop connecting β1 two transmembrane segments.	Gain-of-function mutation rendering enhanced Ca^2+^ sensitivity	Fernandez-Fernandez et al., 2004
*KCNMA1*/BKα	Generalized epilepsy and paroxysmal dyskinesia	D434G/RCK1 domain	Gain-of-function mutation leading to enhanced Ca^2+^ sensitivity	Du et al., 2005; Yang J. et al., 2010
*KCNMA1*/BKα	Epilepsy	N995S/RCK2 domain	D*e novo* mutation Shifts the voltage dependence to more negative potentials without altering Ca^2+^ sensitivity	Li et al., 2018
*KCNMB3*/BKβ3	Idiopathic generalized epilepsy	Del A750/C-terminal region (truncation of 21 amino acids)	BK inactivation	Hu et al., 2003; Lorenz et al., 2007


*This table includes other mutations not linked to neurological diseases that are not discussed in detail in the main text (indicated with asterisks).*

## Neuronal Expression and Functional Roles of K_Ca_ Channels

K_Ca_ channels are expressed in neurons and other cell types in the central nervous system (CNS), where they are involved in a large variety of physiological functions. Due to the vast distribution of K_Ca_ channels and their pleiotropic roles in the CNS, functional studies have been performed in different neuronal types, developmental stages or animal species. It is important to take into account the diversity of these approaches when interpreting the results described in the present review.

Activation of SK channels regulates firing in pacemaker neurons of midbrain and cerebellum, which is critical for muscle coordination and movement (Wolfart and Roeper, 2002; Womack and Khodakhah, 2003). In many neurons, action potentials are followed by a medium-duration after-hyperpolarization (mAHP) that affects their intrinsic excitability (Adelman et al., 2012). In numerous neuron types, the mAHP underlying current (*I*_m_AHP), which can last up to hundreds of milliseconds, is Ca^2+^-dependent but voltage-insensitive and is blocked by apamin but not by BK channel blockers, suggesting the involvement of SK channels (Sah, 1996; Stocker et al., 1999; but see Gu et al., 2005). The three SK channel α subunits, SK1, SK2 and SK3 display characteristics similar to I_m_AHP in heterologous expression systems but fail to fully reproduce the pharmacological and biophysical properties observed *in vivo* (Stocker et al., 1999). This conundrum may be explained by the existence of SK heteromultimeric channels, which have been also described in heterologous expression systems (Ishii et al., 1997a). The idea that SK channels can assemble either as homo- or heteromultimeric channels *in vivo* is supported by their expression pattern in brain, which is overlapping in many regions (Stocker and Pedarzani, 2000). Interestingly, recent evidence shows that SK channels preferentially form species-specific heteromultimers when expressed in HEK cells, by interaction of their C-terminal regions (Tuteja et al., 2010; Church et al., 2015). These authors also show the physiological relevance of SK heteromultimers in CA1 pyramidal neurons (Church et al., 2015) and cardiomyocytes (Tuteja et al., 2010), highlighting the need of further studies to address the implications of heteromeric SK channels in brain and heart.

A role of SK channels in dendritic spines of pyramidal neurons in hippocampus and amygdala has been postulated, where they may regulate the amplitude of excitatory postsynaptic potentials (EPSPs) (Faber et al., 2005; Ngo-Anh et al., 2005; Bloodgood and Sabatini, 2007). During EPSPs, activation of SK channels limits Ca^2+^ influx into the spines and dendrites, thus creating a negative feedback loop controlling EPSPs amplitude. In addition, this activation of SK channels may influence NMDAR receptor activation (via voltage-dependent Mg^2+^ block), consequently affecting synaptic plasticity (Ngo-Anh et al., 2005). In agreement with this, blockade of SK channels in pyramidal neurons from hippocampus (Stackman et al., 2002) and amygdala (Faber et al., 2005; Ngo-Anh et al., 2005) enhanced long-term potentiation (LTP), suggesting that dendritic SK channels may play an important role in memory and learning processes (Stackman et al., 2002).

IK channels are widely expressed throughout the body, excluding some excitable tissues such as cardiac muscle or skeletal muscle. IK channels regulate the membrane potential and Ca^2+^ signaling and are responsible of activation and proliferation of vascular smooth cells and lymphocytes (Toyama et al., 2008; Di et al., 2010; Yu et al., 2013). Immunohistochemical staining experiments indicate that human enteric, sensory and sympathetic neurons also express IK channels (Furness et al., 2004; Bahia et al., 2005; Mongan et al., 2005). In the CNS, IK channels are mainly localized in microglia and endothelial cells (Pedarzani and Stocker, 2008). Microglial IK channels participate in controlling several functions including respiratory burst, proliferation, migration as well as lipopolysaccharide mediated nitric oxide production (Kaushal et al., 2007; Nguyen et al., 2017). Accumulating evidence indicates that IK channels are involved in the process of astrogliosis, which occurs in most forms of CNS insults (Bouhy et al., 2011; Chen et al., 2011; Yu et al., 2014). It has been proposed that, in addition to BK (see below), IK channels also contribute to neurovascular coupling in astrocytes (Longden et al., 2011).

In many neuron types including hippocampal CA1 and CA3 pyramidal cells, repetitive firing stimulus elicits a slow, long lasting hyperpolarization, which is referred to as slow AHP (sAHP) (Adelman et al., 2012). The development of sAHP prevents the generation of new action potentials, limiting hyperexcitation of neurons in response to a long lasting or repetitive stimulus. Several studies have suggested that both SK (Lima and Marrion, 2007) and Kv7 channels (Tzingounis and Nicoll, 2008; Tzingounis et al., 2010) may contribute to sAHP. However, this assertion is contradicted by other observations showing that blockers of those channels produce only a minor reduction of the sAHP current (I_s_AHP), suggesting that other channels are involved (Womble and Moises, 1993; Power and Sah, 2008). Furthermore, genetic ablation of SK1, SK2 or SK3 does not affect sAHP in CA1 pyramidal neurons (Bond et al., 2004). Although IK channels have been suggested to underlay sAHP (King et al., 2015), a recent study ruled out this hypothesis based on the pharmacological properties of I_s_AHP, which were unaltered in IK knockout mice (Wang K. et al., 2016). The molecular identity of the sAHP channels remains unknown.

BK channels are localized throughout the dendrites, axon, soma and synaptic terminals of neurons in different brain regions, including cortex and hippocampus (Knaus et al., 1996; Grunnet and Kaufmann, 2004; Wang et al., 2014), where they play key roles in action potential duration, firing frequency and neurotransmitter release (for a recent review, see Contet et al., 2016). It should be noted that depending on the subcellular location where they are expressed, BK channel isoforms may exhibit different biophysical and trafficking properties, determined by the subunit composition (assembly with auxiliary subunits) and alternative splicing (Contet et al., 2016). At the synaptic terminals, it has been proposed that tight spatial coupling between BK channels and VGCCs (Marrion and Tavalin, 1998) constitutes a molecular brake for neurotransmitter release, by the BK-mediated termination of action potentials (Bielefeldt and Jackson, 1993; Hu et al., 2001; Muller et al., 2007; Contet et al., 2016). In dendrites, this association is also relevant, contributing to regulation of the magnitude and duration of dendritic Ca^2+^ spikes to influence action potential output (Engbers et al., 2013; Indriati et al., 2013). BK channels have the ability to shape the action potential waveforms and influence the frequency of firing in different ways. Pharmacological or genetic deletion of BK channels can increase or decrease the evoked or spontaneous firing frequency (Bielefeldt and Jackson, 1993; Nelson et al., 2003; Gu et al., 2007). Many studies have shown that blockade of BK channels using TEA, charybdotoxin or paxilline slows action potential repolarization and reduces the magnitude of the fast-duration AHP (Adams et al., 1982; Storm, 1987; Sah and McLachlan, 1992; Zhang and McBain, 1995; Faber and Sah, 2002). In the suprachiasmatic nucleus, BK channels contribute to spontaneous firing rate related to normal circadian behavioral rhythm (Meredith et al., 2006; Whitt et al., 2016; Whitt et al., 2018). Genetic ablation of the BKβ_4_ subunit in neurons from the dentate gyrus (DG) augmented the fast AHP amplitude, sharpened the action potential and increased spike frequency (Brenner et al., 2005). These findings support the idea that BK channels are not strictly excitatory or inhibitory but can be dynamically regulated to control neuronal excitability (Contet et al., 2016). Regulatory mechanisms include coupling to accessory subunits (Brenner et al., 2005; Whitt et al., 2016) and different sources of intracellular Ca^2+^ (Wang B. et al., 2016; Whitt et al., 2018).

Expression of BK has been also reported in astrocytes (Seidel et al., 2011; Seifert et al., 2018), where they have been related to astrocytic function connecting neuronal signaling to CNS vasculature (Howarth, 2014). Many studies have highlighted the role of astrocytic processes (endfeet) in modulating the vascular response by dynamically controlling intracellular Ca^2+^ (Filosa et al., 2006; Takano et al., 2006; Girouard et al., 2010). It has been proposed that activation of BK channels by elevated Ca^2+^ in astrocytic endfeet leads to vasodilation by the release of K^+^ into the perivascular space (Price et al., 2002; Filosa et al., 2006). Interestingly, another study demonstrated that this mechanism can also mediate vasoconstriction, leading the authors to propose that BK channels may produce dual vascular responses depending on the Ca^2+^ levels at the astrocytic endfoot (Girouard et al., 2010).

### Neuroprotective Roles of K_Ca_ Channels

Dopamine (DA) neurons in the midbrain retrorubral field (RRF), substantia nigra *pars compacta* (SNc) and ventral tegmental area (VTA) influence the neurons in target areas due to their extensive axonal projections (Matsuda et al., 2009). Thus, DA neurons are involved in regulating essential processes such as fine motor control (Bernheimer et al., 1973) learning and memory (Matsumoto and Takada, 2013), cognitive processes (Nieoullon, 2002), response to stress (Ungless et al., 2010) and noxious stimuli (Brischoux et al., 2009). SK channels control the pattern of single spike firing of DA neurons both *in vivo* and *in vitro*, as demonstrated by the reduction in firing regularity observed after treating the cells with apamin (Waroux et al., 2005; Ji and Shepard, 2006). Some studies have postulated that SK channels may exert neuroprotective effects against progressive DA neuronal death (Dolga et al., 2014), by a proposed mechanism in which activation of SK channels in SNc neurons counteracts excitotoxicity associated to NMDAR overactivation (Dolga et al., 2014; Duda et al., 2016). Additionally, SK channels could directly regulate NAPDH oxidase function, thus hampering neuronal damage by preventing production of reactive oxygen species (ROS) in mitochondria (Fay et al., 2006; Dolga et al., 2014). In mouse CA1 pyramidal neurons, a protective role of SK2 channels has been described, based on the observation that direct activation of SK channels by 1-ethyl-2- benzimidazolinone (1-EBIO) significantly reduced neuronal death in a model of cerebral ischemia induced by cardiac arrest/cardiopulmonary resuscitation. This finding points to activation of SK channels as a potential neuroprotective target in cerebral ischemia (Allen et al., 2011).

Accumulating evidence supports multifaceted functions of IK channels (Kaushal et al., 2007; Dolga and Culmsee, 2012). Specifically, IK channels have been related to microglia activation by modulating Ca^2+^ signaling, oxidative burst, proinflammatory cytokines production and microglia-mediated neuronal cell death (Kaushal et al., 2007). According to these proposed physiological roles, a recent study demonstrated that genetic ablation or *in vivo* pharmacological blockade of IK channels in the middle cerebral artery occlusion (MCAO) mouse model reduced the infarct area as well as improved neurological deficit by reducing microglia-associated neuronal death (Chen Y.J. et al., 2016).

A protective role of BK channels in the context of acute focal cerebral ischemia has been tested *in vivo* by performing MCAO in BK knockout mouse models, which showed higher post-ischemic mortality than their WT littermates (Liao et al., 2010). In this context, it was suggested that BK plays a neuroprotective role by counteracting NMDA-induced neurotoxicity, since genetic silencing or blockade of BK channels in organotypic hippocampal slice cultures (Liao et al., 2010) or cortico-striatal brain slice cultures (Katsuki et al., 2005) showed higher levels of NMDA-induced neuronal death when they were exposed to ischemic-like conditions.

K_Ca_ channels are also expressed at inner mitochondrial membranes (mitoK_Ca_), where they have been suggested to be involved in neuroprotection by different mechanisms, some of which are still unknown (Bednarczyk, 2009). The activation of mitoSK channels in conditions of glutamate toxicity enhanced the mitochondrial resilience and reduced cell death in mouse hippocampal cell lines (Dolga et al., 2013; Honrath et al., 2017). Additionally, mitoBK channels were found to preserve neuronal cell viability by attenuating the overall ROSs production (Kulawiak et al., 2008; Skalska et al., 2009). In addition to these reports, several others have reviewed the importance of mitoK_Ca_ channels as potential targets for neuroprotection (Szewczyk et al., 2010; Balderas et al., 2015; Krabbendam et al., 2018).

## Role of K_Ca_ Channels in Neurological and Psychiatric Diseases

Concomitant with their physiological roles, alterations in K_Ca_ function lead to excitability disorders that are characterized by abnormal firing of neuronal networks. The advancement of genetic linkage analysis over the past decades significantly facilitated the identification of specific loci associated to disease, many of which include genes encoding K_Ca_ channels subunits (Cooper and Jan, 1999; see **Table 1**). The role of these channels in disease has been also addressed using pharmacological tools. In this section we review the proposed contribution of BK and SK channels to neurological diseases. It is worth noting that, to our knowledge, no human diseases have been described in the literature involving K_Ca_3.1 (IK) channel mutations. Their potential as drug targets in neurodegenerative diseases and ischemic stroke is reviewed in latter sections referring to K_Ca_ modulators.

### Role of SK Channels

Malfunction of SK channels has been related to epilepsy. In pilocarpine-treated epileptic rats, the expression and function of SK channels was significantly reduced (Oliveira et al., 2010). Using thalamic slices treated with bicuculline methiodide as an experimental model of absence epilepsy, Kleiman-Weiner et al. (2009) found that blockade of SK channels modulated epileptiform oscillations by reducing intrinsic excitability of reticular neurons with no effect on relay neurons, pointing to SK channels as potential therapeutical targets for this disease. The involvement of SK in epilepsy has been further studied by using different modulators (see following section in this review).

Schizophrenia is a progressive neurodegenerative disorder that is characterized by disintegration of processes involved in thinking and emotional responsiveness (Postmes et al., 2014), that has been linked to DA imbalance (Brisch et al., 2014). A spontaneous mutation of the SK3 channel gene (*L283fs287X*) resulting in the deletion of the protein N-terminal region was identified in human schizophrenia patients (Bowen et al., 2001) and the resulting altered channels (hSK3Δ) were found to dominantly suppress SK channel currents. (Miller et al., 2001). Selective expression of this SK3 mutant in mice DA neurons additionally reduced the coupling between the SK channels and NMDAR, leading to increased burst firing as well as amplification of DA release, ultimately impacting animal behavior (Soden et al., 2013). The results by Soden et al. (2013) additionally showed that functional deficits of SK channels can alter the balance between phasic and tonic DA signals, which are normally associated to the pathogenesis of schizophrenia (Grace, 1991).

Pathogenesis of Parkinson’s disease (PD), which is associated to death of DA neurons in the SNc (Surmeier et al., 2010) has been also related to K^+^ channels dysfunction, including SK channels (Wang et al., 2008; Chen et al., 2018). This hypothesis is consistent with the observed roles of SK channels as regulators of dopaminergic neurotransmission in the SNc, protecting DA neurons from excitotoxic death (as reviewed in the previous section; see also Dolga et al., 2014). However, the role of SK channels in the etiology of PD remains elusive due to contradictory evidence. Some reports showed that activation of SK channels by NS309 in human DA neurons inhibited spontaneous firing, enhancing mAHP (Ji and Shepard, 2006), and reducing neurotoxicity (Dolga et al., 2014). These results suggest that increasing SK channel activity could boost or at least preserve levels of DA synthesis, which would in turn mitigate the long-term motor symptoms of PD. On the other hand, several studies have provided evidence that blockade of SK channels by apamin improved the symptoms of PD *in vitro* and *in vivo* (Doo et al., 2010; Kim et al., 2011; Alvarez-Fischer et al., 2013). This idea was further strengthened by the fact that blocking SK channels restored minimal DA activity in the striatum, alleviating the non-motor symptoms induced by partial striatal DA lesions (Chen et al., 2014). A possible interpretation of these apparently contradictory results is that SK channel positive or negative modulators could be beneficial depending on the stage (early or late) of the disease.

### Role of BK Channels in Neurological Diseases

BK malfunction has been related to seizures and epilepsy, although the role of these channels in this pathological condition seems to be complex. *KCNMA1* knockout mice did not show spontaneous seizures, suggesting that CNS excitability is unaffected and discarding a prominent role of BK hypoactivity in epileptogenesis (Sausbier et al., 2004). However, growing evidence associates BKα gain-of-function or loss-of-function to neuronal pro-excitatory or pro-inhibitory effects, which may depend on the cellular context (for recent reviews, see (N’Gouemo, 2014; Contet et al., 2016). A BKα gain-of-function mutation (D434G) showing larger macroscopic currents, increased intrinsic gating and Ca^2+^ sensitivity was associated with a human syndrome of generalized epilepsy and paroxysmal dyskinesia (Du et al., 2005). Studies addressed to characterizing the mechanism underlying the strong functional impact of this mutation have greatly contributed to a better understanding of the biophysics of BK channels. Since the D434 site is located in the vicinity of the RCK1 Ca^2+^ binding site of the BK channel, its alteration was found to affect the allosteric coupling between the Ca^2+^ binding and the opening of the channel (Yang J. et al., 2010). Enhanced neuronal BK channel activity as a result of the D434G mutation would increase the fAHP, resulting in faster recovery of sodium channels from inactivation, enabling neuronal firing at higher frequency and ultimately leading to increased neuronal excitability and seizures (Du et al., 2005; Diez-Sampedro et al., 2006; Wang et al., 2009; Yang J. et al., 2010). In a recent study, Li et al. (2018) identified a *de novo* mutation in the BKα gene (N995S) associated with epilepsy but not with paroxysmal dyskinesia. In contrast to the D434G mutant, the N995S gain-of-function variant shows alterations of the channel’s sensitivity to voltage, but not to Ca^2+^ (Li et al., 2018). This notion that hyperactivity of BK channels is related to hyperexcitability and seizures is further supported by their implication in pathogenesis of alcohol withdrawal seizures (Ghezzi et al., 2014). Furthermore, blockade of BK has been shown to reduce neuronal hyperexcitability in animal models of epilepsy and seizures, but does not present anticonvulsant effects in animals with no previous seizure episodes (Jin et al., 2000; Sheehan et al., 2009). BK pro-excitatory or pro-inhibitory effects can be additionally determined and modulated by association to regulatory subunits. A recent study by Whitmire et al. (2017) showed that β_4_ expression in hippocampal DG granule neurons is downregulated following pilocarpine-induced seizures, leading to gain-of-function BK and increased excitability. This result is consistent with previously observed effects in β_4_ knockout mice (Wang et al., 2009; Whitmire et al., 2017). Alteration of the BKβ_3_ regulatory subunit as a consequence of a single base pair deletion (delA750) in the *KCNMB3* gene has also been associated to idiopathic generalized epilepsy in humans (Lorenz et al., 2007). This variant confers loss-of-function characteristics to BKα in heterologous expression systems by enhancing inactivation (Hu et al., 2003).

Two independent genome-wide association studies reported that single nucleotide polymorphisms in the *KCNMA1* gene encoding the BKα subunit (Beecham et al., 2009) and in the *KCNMAB2* gene (regulatory subunit BKβ_2_) (Beecham et al., 2014) are strongly linked to the pathophysiology of Alzheimer’s disease (AD). Intracellular application of amyloid β peptide (*β*) in neocortical pyramidal neurons from rats and mice increased neuronal excitability by reducing function of BK channels (Yamamoto et al., 2011). In AD and depression mice models, transcranial magnetic stimulation (TMS) reduced the depression-like behavior by a proposed mechanism in which the activation of BK channels by TMS would enhance hippocampal LTP and inhibit cortical excitability (Sun et al., 2011; Wang F. et al., 2015). Interestingly, TMS was found to improve cognitive functions in human AD patients (Cotelli et al., 2006; Ahmed et al., 2012).

BK malfunction has been also related to Fragile X syndrome (FXS), a monogenic form of intellectual disability and autism, associated to transcriptional silencing of the *Fmr1* gene encoding Fragile X mental retardation protein (FMRP) (Verkerk et al., 1991). Recent evidence has shown that FMRP regulates neurotransmitter release in CA3 neurons by directly interacting with BKβ4 subunits (Deng et al., 2013). Consistent with this hypothesis, Hebert et al. (2014) restored the FXS behavioral deficits in Fmr1 knockout mice by treating the animals with selective BK channel openers (see below). These results support the idea that BK channels may be potential therapeutic targets for FXS (Brager and Johnston, 2014; Contractor et al., 2015).

Habituation is a form of learning in which the behavioral response to a stimulus decreases following repeated exposure to the stimulus (Rankin et al., 2009). Habituation to the acoustic startle response is impaired in many psychiatric disorders including schizophrenia and autism (Braff et al., 1992; Ornitz et al., 1993). BK channels have been implicated in habituation by controlling synaptic transmission (Typlt et al., 2013). Recently, it has been shown that activation of BK channels and their subsequent phosphorylation are essential for synaptic depression underlying habituation (Zaman et al., 2017).

## Regulation of K_Ca_ Channels and Implications for Therapeutic Approaches

**FIGURE 2:**
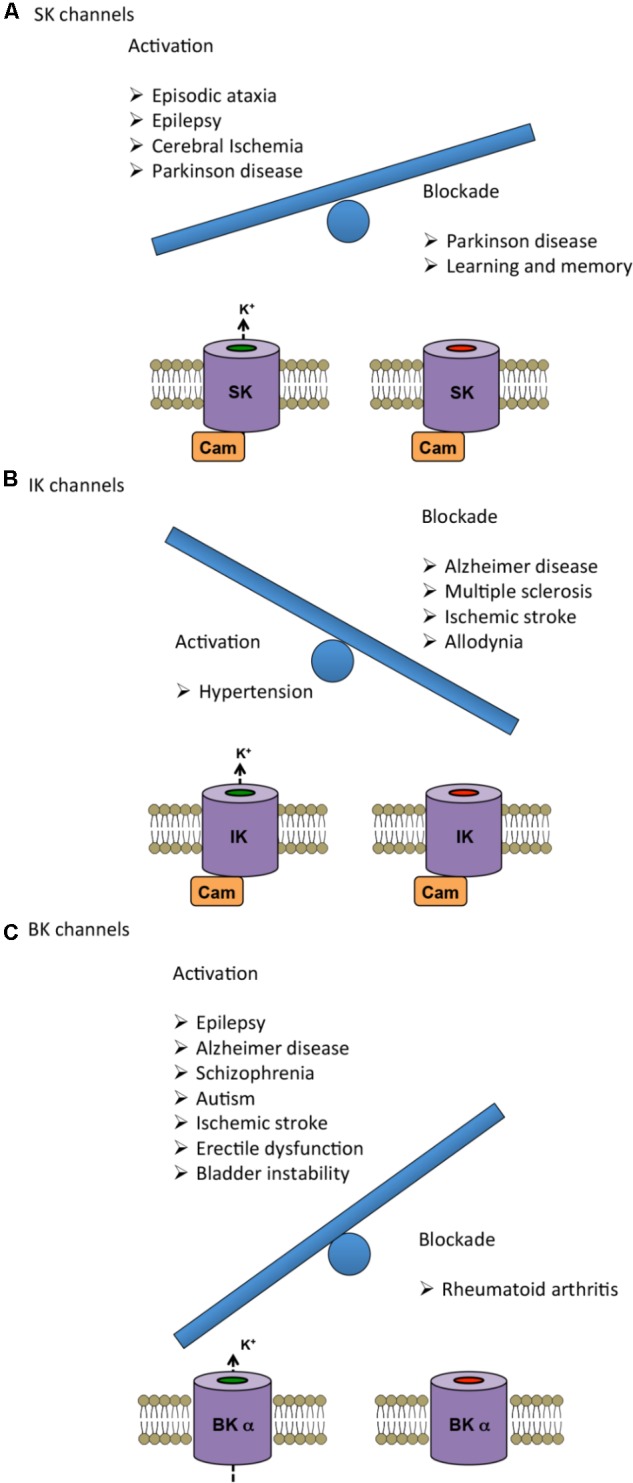
Therapeutic benefits of either activation (left) or blockade (right) of K_Ca_ channels represented as a weighing scale. The heavier balance indicates the modulation (activation or blockade) that has been reported advantageous in a larger number of diseases. **(A,C)** In the case of SK and BK channels, activation shows more beneficial effects than inhibition. **(B)** For IK channels, pharmacological blockade has proven to be more advantageous than activation. For further information about the specific effects, see Tables and main text.

As described in the previous sections, alterations in K_Ca_ channels are associated to several genetically linked and acquired diseases. In this section we review the use of various K_Ca_ modulators oriented toward disease treatment (summarized in **Table 2**), including some pharmacological considerations. The current state-of-the-art knowledge on the potential of K_Ca_ channels as therapeutic targets is summarized in **Figure 2** by representing as a weighing scale the beneficial effects resulting from either activation (left side) or blockade (right side) of each K_Ca_ subtype. Interestingly, SK and BK channels’ balance is tipped toward the activation side, which seems to be more beneficial than blockade. Conversely, for IK channels blockade is more favorable than activation.

**Table 2:** Functional roles of clinically available/potential modulators of K_Ca_ channels.

Target	Drug	Reported effects	Comments	References
SK	1-EBIO	-Positive effects on cerebral ischemia animal models	-SK activator	Allen et al., 2011; Lappin et al., 2005; Anderson et al., 2006; Oliveira et al., 2010
		-Reduces epileptiform activity in an acute model of epilepsy		
	NS309	Positive effects on DA neurons function related to Parkinson’s disease	-SK activator	Ji and Shepard, 2006; Dolga et al., 2014
	Chlorzoxazone	Improves motor-coordination and other symptoms of episodic ataxia type-2	-SK activator	Alvina and Khodakhah, 2010
			-FDA-approved treatment for spasticity	
	Riluzole	-Positive effects in arthritic pain model	-SK activator	Ristori et al., 2010; Thompson et al., 2015
		-Improves ataxia-related symptoms in human patients	-FDA-approved treatment for ALS	
	DCEBIO	Reduces recall of extinction memory in rats	SK activator	Criado-Marrero et al., 2014
	Apamin	-Improves Parkinson’s disease symptoms *in vitro* and *in vivo*	SK inhibitor	Deschaux et al., 1997; Inan et al., 2000; Stackman et al., 2002; Mpari et al., 2005; Doo et al., 2010; Yang E.J. et al., 2010; Kim et al., 2011; Alvarez-Fischer et al., 2013; Criado-Marrero et al., 2014
		-Positive effects on various memory deficits and spatial learning		
IK	TRAM-34	-Positive effects in AD, ischemic stroke and multiple sclerosis mouse models	IK inhibitor	Reich et al., 2005; Chen Y.J. et al., 2016; Yi et al., 2016; Lu et al., 2017
		-Increases formalin-induced nociceptive behavior		
	Senicapoc	-Positive effects in sickled cell anemia disease (SAD)^∗^	-IK inhibitor	Wulff and Castle, 2010; Staal et al., 2017
		-Reversed tactile allodynia in rats with peripheral nerve injury	-Phase 3 clinical trials for SAD	
			-Phase 2 clinical trials for asthma	
BK	Isoprimaric acid (ISO)	-Improves cognitive defects in AD animal models	-BK activator	Wang L. et al., 2015
	GoSlo	-Inhibits spontaneous contractions in both bladder and corpus cavernosum smooth muscle tissues.	-BK activator	Large et al., 2015; Hannigan et al., 2017
	Andolast	Positive effects on asthma treatment	-BK activator	Malerba et al., 2015
			-Currently in late stage of phase 3 clinical trials for asthma	
	BMS204352	-Positive effects on FXS animal models	-BK and Kv7 activator	Jensen, 2002; Brown and Passmore, 2009; Hebert et al., 2014; Zaman et al., 2017; Carreno-Munoz et al., 2018
		-Enhances short-term habituation	-Phase 3 clinical trials for treatment of acute ischemic stroke^∗^	
		-Positive effects on spontaneous hypertensive rats model^∗^		
	Zonisamide	-Positive effects on a wide range of epilepsies and neuropsychiatric disorders	-BK activator, Na^+^ and Ca^2+^ channel modulator	Biton, 2007; Huang et al., 2007
			-Antiepileptic drug used clinically	
	Iberiotoxin (IbTX)	Beneficial effects in rheumatoid arthritis animal models	selective BKαβ_1-3_ blocker	Tanner et al., 2018


*This table includes additional information, not detailed in the main text, about K_*Ca*_ modulators reported in the literature to treat diverse pathological conditions.*

### Modulators of SK Channels

Several modulators of SK function have been identified as the basis for potential treatment of neurological diseases. Chlorzoxazone (CZX) is a SK channel activator, which has been approved by the U.S. Food and Drug Administration (FDA) as a muscle relaxant to treat spasticity (Losin and McKean, 1966). In addition, it has been proposed to be a viable pharmacotherapeutic approach for episodic ataxia type-2 (EA2) treatment. Oral administration of CZX in an EA2 mouse model improved motor coordination and reduced the severity, frequency and duration of dyskinesia episodes with no side effects, by a proposed mechanism of restoring SK channels function (Alvina and Khodakhah, 2010).

Riluzole is the first FDA-approved treatment for amyotrophic lateral sclerosis (ALS) (Miller et al., 2012). Although the molecular target of riluzole remains elusive, many studies have proposed that it works by modulating SK channels (Dimitriadi et al., 2013; Lam et al., 2013). In spinal muscular atrophy animal models, treatment with riluzole ameliorated disease-related loss-of-function defects by acting on SK channels (Dimitriadi et al., 2013). A recent study demonstrated that riluzole inhibits supraspinally organized pain behavior in a rat model of arthritic pain by activating SK channels (Thompson et al., 2015). Beneficial effects of riluzole have also been reported for the treatment of spinocerebellar ataxia type 2 (SCA2), a disease involving irregular firing and eventual neuronal death of Purkinje cells (PC) (Kasumu and Bezprozvanny, 2012). Administration of riluzole in patients improved ataxia-related symptoms (Ristori et al., 2010). This result was paralleled in a SCA2 transgenic mouse model, in which treatment with the SK selective activator NS13001 alleviated the symptoms of the disease (Kasumu et al., 2012). The mechanism underlying the effect of riluzole seems to reside in the activation of SK2 channels predominant in PC (Durr, 2010), which has been implicated in regulation of tonic firing (Ito, 2002; Walter et al., 2006). Consistently with this hypothesis, Walter et al. (2006) observed an improvement in motor coordination as well as restored PC firing regularity after treatment of an ataxia mouse model with SK channel modulators.

Selective activation of SK channels could be an effective therapy in some epileptic conditions. Potentiation of SK channels using ethylbenzimidazolinone (EBIO) was shown to inhibit epileptiform bursting in rat hippocampal CA3 neurons (Lappin et al., 2005). Additionally, this activator also reduced seizure incidence following maximal electroshock and increased the threshold to pentylenetetrazole-induced seizures in mice (Anderson et al., 2006).

*In vivo* infusion of SK activator 5,6-Dichloro-1-ethyl-1,3-dihydro-2*H*-benzimidazol-2-one (DCEBIO) in rats reduced recall of extinction memory, leading the authors to propose SK as a therapeutic target in anxiety disorders (Criado-Marrero et al., 2014). However, this result also suggests that the effect of SK activators on learning and memory should be taken into account in potential therapies.

The use of SK channel blockers has been also explored for disease treatment. Initial studies showed that SK blockers are not neuroprotective and might detrimentally accelerate neurodegeneration (Mourre et al., 1997). However, administration of apamin in rats and mice accelerated spatial learning (Deschaux et al., 1997; Stackman et al., 2002), promoted object recognition memory (Mpari et al., 2005) and facilitated the recall of extinction memory (Criado-Marrero et al., 2014). Additionally, apamin also mitigated the memory deficits associated to scopolamine- or electroconvulsive shock-induced amnesia (Inan et al., 2000). In summary, SK activators and blockers seem to be involved in distinct beneficial effects that would need to be balanced in order to design specific therapies.

### Modulators of IK Channels

Consistent with the physiological roles of IK channels described in previous sections of this review, the selective inhibitor TRAM-34 (1-[(2- chlorophenyl)diphenylmethyl]-1*H*-pyrazole) decreased astrogliosis and microglial activation and attenuated memory loss in an AD mouse model (Yi et al., 2016). Furthermore, reduced microglia activation after blocking IK channels in an ischemic stroke mouse model has also been documented, which resulted in reduced infarction size and improved neurological deficit (Chen Y.J. et al., 2016). Additionally, application of TRAM-34 alleviated the symptoms of experimental autoimmune encephalomyelitis, a murine model of multiple sclerosis (Reich et al., 2005). Together, these data suggest that pharmacological blockade or genetic deletion of IK channels may represent a therapeutic target for various neurodegenerative disorders and ischemic stroke. Nonetheless, it is important to point out that although pharmacological blockade of IK channels appeared to be safe and well tolerated in animal models and humans, the genetic deletion induced an increase in blood pressure (Si et al., 2006). On the other hand, no study has yet reported increased blood pressure with IK channel blockers including treatment of mice with TRAM-34 (Toyama et al., 2008) or human volunteers taking Senicapoc (Ataga et al., 2008). Finally, recent evidence suggests that IK inhibitors should be used with caution since they may also increase tonic pain in certain clinical conditions. A recent study demonstrated that pharmacological inhibition of IK channels using TRAM-34 in mice increased formalin-induced nociceptive behavior (Lu et al., 2017). Conversely, another study showed that blocking IK channels with Senicapoc reversed tactile allodynia in rats with peripheral nerve injury (Staal et al., 2017).

### Modulators of BK Channels

Zonisamide is an antiepileptic drug used clinically as adjunctive therapy in refractory partial-onset seizures that has also shown beneficial effects in a wide range of epilepsies and neuropsychiatric disorders (Biton, 2007). In addition to inhibiting neuronal Na^+^ and Ca^2+^ ion channels, Huang et al. showed that zonisamide activated BK channels in a hippocampal neuron-derived cell line, suggesting that the effects of zonisamide may be mediated, at least partly, by modulation of these channels (Huang et al., 2007).

A recent study suggests that BK channel activators could have therapeutic potential for cognitive defects in AD patients. Chronic application of the BK channel activator isoprimaric acid (ISO) enhanced non-spatial memory in AD mice, restoring the basic synaptic transmission and LTP at the hippocampal CA1 synapses, which were elevated and suppressed respectively in the disease model (Wang L. et al., 2015). Interestingly, ISO treatment not only enhanced BK channel activity but also reduced the Aβ fraction suggesting that a two-way relationship exists between BK channels and the Aβ plaque (Wang L. et al., 2015).

Treatment of *FMR1* knockout mice, an animal model of FXS, with the selective BK channel opener BMS-204352 reversed the abnormal dendritic spine phenotype *in vitro.* Additionally, direct injection of the activator restored hippocampal glutamate homeostasis and corrected disturbances in social recognition and interaction, non-social anxiety and spatial memory (Hebert et al., 2014). In another study, BMS-204352 efficiently reversed the cortical hyperexcitability and increased acoustic startle seen in *FMR1* knockout mice (Carreno-Munoz et al., 2018), suggesting that this treatment could alleviate sensory hypersensitivity symptoms in FXS patients.

Finally, BK channel openers have been tested to restore impaired habituation associated to some psychiatric disorders. Systemic administration of BMS-204352 greatly attenuated synaptic depression in the sensorimotor neurons of the caudal pontine reticular nucleus in the brain stem, which was associated with enhanced short-term habituation (Zaman et al., 2017).

## Molecular Strategies and Current Challenges to Target K_Ca_ Channels in Drug Discovery

Ion channels are expressed at distinct physiological niches in the human body where they mediate diverse functions. Although numerous drugs are available to modulate K^+^ channels, currently no K_Ca_ modulators are clinically used. One potential reason for this comparative failure in the drug discovery process relates to the fact that the study of channel complexes in physiological conditions is challenging, due to their intricate functional mechanisms. For instance, the diversity of physiological roles fulfilled by BKα channels in neurons is greatly enhanced by coexpression with tissue specific auxiliary β subunits, which confer tissue- and subcellular-specific characteristics to the BK currents determining the action potential shape, firing rate and pharmacology (Jaffe et al., 2011; Wang et al., 2014; Whitt et al., 2016). This otherwise beautiful example of evolutionary complexity imposes an arduous task on the drug designing process, which must specifically target the various subunit compositions. Although some encouraging results may have been obtained in heterologous expression systems, quite often the potential compounds modulating K_Ca_ channels are less potent *in vivo*, leading to undesirable side effects. This section reviews some considerations about the existing technical approaches to test the efficiency and efficacy of potential modulators, as well as the use of other structural and genetic approaches to improve targeting of specific K_Ca_ channel subunits to particular tissues.

Electrophysiological testing of ion channel function on heterologous expression systems still remains the backbone for functional validation of potential modulators. However, the major downside of both conventional and automated electrophysiology is that throughput efficiency is limited and operational costs are high, so there is a significant need for the development of novel robust, affordable and effective high-throughput screening methods (Picones et al., 2016). Membrane potential-based drug assays constitute a robust method for assessing the K^+^ channel response to numerous drug candidates, although they seem to be subject to a large number of false positives (Solly et al., 2008). Ion flux assays are useful tools for screening compounds in drug discovery processes (Gill et al., 2003) including BK channel modulators (Jorgensen et al., 2008, 2013). This approach utilizes thallium or rubidium radioisotopes as K^+^ ion substitutes providing sensitive, precise and reproducible results, yet with some limitations related to the radioactivity and toxicity of these ions. A novel and imaginative cell free assay is the liposome flux assay (LFA), where purified K^+^ channels are reconstituted into lipid vesicles from which K^+^ flux is recorded and optically monitored with a high signal-to-noise ratio. Su et al. (2016) demonstrated the robustness of this technique by screening a library of 300,000 compounds targetting K^+^ ion channels, including BK.

Structural data is an essential tool supporting and complementing the discovery of ion channel-modulating drugs by providing information about drug–protein binding interactions. This knowledge is in turn necessary to improve the potency, efficacy and selectivity of the candidate drug compounds. Until very recently, available SK crystal structures were restricted to the Ca^2+^-gating domains (CaM and CaMBD) (Schumacher et al., 2001, 2004; Li et al., 2009; Zhang et al., 2012a,b, 2013; Zhang M. et al., 2014; Nam et al., 2017), lacking information about the transmembrane domains, therefore making the structure-based drug discovery a complicated task. Based on available structural information, some positive modulators of SK channels including PHU, 1-EBIO and NS309 were proposed to bind to the CaM/CaMBD interface (Strobaek et al., 2004; Zhang et al., 2012b, 2013; Morales et al., 2013; Brown et al., 2017; Nam et al., 2017). However, these conclusions are now challenged by the cryo-EM full-length IK-CaM structures only recently unveiled by the MacKinnon laboratory, which redefine the native interface between the CaM N-lobe and the C-terminal end of S6, showing a predominant role of the S4-S5 linker in Ca-CaM mediated channel gating (Lee and MacKinnon, 2018). Previous considerations should be revisited in light of these novel findings, which undoubtedly set an unprecedented ground for SK channels drug discovery. These new IK structures appear barely 1 year after the cryo-EM structures of the full-length BK channel in the open and closed states was obtained (Hite et al., 2017; Tao et al., 2017), revealing new and interesting insights in the channel structure–function relationships (Giraldez and Rothberg, 2017; Zhou et al., 2017; Kshatri et al., 2018). In this context, other techniques such as patch-clamp fluorometry (Zheng and Zagotta, 2003; Wulf and Pless, 2018) have proven to be useful tools to study conformational changes between subunits at the level of the gating ring during activation of functional BK channels (Giraldez et al., 2005; Miranda et al., 2013, 2016), and could also be extended to study other members of the K_Ca_ subfamily. Altogether, these approaches are revolutionizing our knowledge about how these ion channels can be selectively drug-targeted.

Developing specific rodent disease models based on characterized mutations has become a standard procedure to understanding their physiological relevance. In most cases, these models recapitulate the disease phenotypes and are useful as probing tools to characterize the channel’s cellular and neuronal functions. Furthermore, they might constitute preclinical tools to attain a better understanding of the disease-causing mechanisms, consequently helping to build therapeutic strategies targeting the disease. The development of the CRISPR genome editing technique has facilitated generation of disease models by introducing loss- or gain-of-function mutations, allowing to study the effects of underlying genetic aberrations (Chen S. et al., 2016).

In addition, taking advantage of novel molecular techniques, new strategies have been developed to target the mutated genes *in vivo*. One example consists on the administration of antisense oligonucleotides to reduce the levels of mutant proteins that could be responsible for the neurodegenerative diseases. In an animal model of ALS caused by a mutation in the SOD1 protein, direct delivery of antisense oligonucleotides significantly slowed disease progression (Smith et al., 2006). In this context, the CRISPR technique undoubtedly constitutes a promising molecular approach to reverse the alterations in proteins and restore their physiological activity. Cheng et al. (2013) demonstrated that injection of CRISPR components into mouse zygotes efficiently activated several endogenous genes. The application of these techniques to target K_Ca_ channels and associated pathologies needs further exploration.

A strategy that has progressed toward clinical trials is gene-based therapy. The first and only phase I trial using this approach was performed on human patients with erectile dysfunction (ED). Plasmids encoding BK channels were injected directly into the penis corpus cavernosum of patients with ED, rendering positive encouraging results, suggesting that gene therapy represents a valuable treatment for ED (Melman et al., 2006, 2007) and could be considered for other K_Ca_-related pathologies.

Finally, combination therapies that integrate tissue delivery of genes and drugs has been shown to be a viable approach enhancing drug specificity (Chen J. et al., 2016). This is especially relevant in the case of BK channels, which are expressed ubiquitously throughout the body, so systemic drug administration often results in off-target effects (Lappin et al., 2005).

## Conclusion and Future Perspectives

K_Ca_ channels are relevant determinants of neuronal excitability and their dysfunction is implicated in many inherited and acquired neurological and psychiatric diseases, thus constituting appealing targets for pharmacological intervention. Over the last few years, the drug discovery field has significantly advanced to identify lead compounds for K_Ca_ channel modulators. Available therapeutic strategies to target these channels are challenging due to their diverse physiological roles and tissue distribution, so the design of modulators that recognize tissue-specific subunit combinations is of special relevance. Structural biology has dramatically advanced over the last decade to reveal many ion channel structures with atomic resolution, including the long-awaited structure of the full-length BK channel and of the human IK-CaM complex. Knowledge of genetic- and drug-induced regulation of K_Ca_ channels coupled to investigation of their structure–function relationships will facilitate the development of disease-specific treatment strategies.

## Author Contributions

AK, AG-H, and TG designed and wrote the manuscript.

## Conflict of Interest Statement

The authors declare that the research was conducted in the absence of any commercial or financial relationships that could be construed as a potential conflict of interest.
